# 

**Published:** 1799-08

**Authors:** 


### THE MEDICAL AND PHYSICAL JOURNAL;

CONTAINING

THE EARLIEST INFORMATION

ON SUBJECTS OF

*Medicine, Surgery, Pharmacy, Chemiſtry,*

AND

NATURAL HISTORY,

AND A CRITICAL ANALYSIS OF ALL NEW BOOKS IN THOSE DEPARTMENTS OF LITERATURE.

CONDUCTED BY

T. BRADLEY, M.D.

AND

A. F. M. WILLICH, M.D.

5708

—— Ex medicina nihil oportet putare proficiſci, niſi quod ad utilitatem corporis ſpectat, quoniam ejus causâ eſt inſtituta.

CICERO, *de Inventione*, Lib. I.

VOL. II.

FROM AUGUST TO DECEMBER, 1799.

*LONDON:*

Printed by WILLIAM THORNE, Red Lyon Court, Fleet Street,

For R. PHILLIPS, No. 71, ST. PAUL'S CHURCH-YARD.

[ Entered at Stationers Hall. ]

### ADVERTISEMENT.

THE Editors have ſo many opportunities of addreſſing their literary friends, correſpondents, and the public, that the formality of a Preface might well be diſpenſed with: they are, however, happy to avail themſelves of this occaſion, to expreſs their ſenſe of gratitude for the numerous and conſtant proofs of the general approbation with which their labours are received.

The increaſing demand for the MEDICAL AND PHYSICAL JOURNAL, ſince the completion of the Firſt Volume, as well as the unprecedented ſupport by original communications, are the moſt unequivocal teſtimonies of public ſanction.

The Conductors of the work embrace this opportunity of aſſuring the profeſſional Reader, that the frequent hints they have received, concerning the protraction of controverſies, and inſertion of anonymous communications, ſhall not be neglected. Indeed, the public mind ſoon decides on conteſted points, if at all intereſted in the inquiry; while the Diſputants themſelves generally become more and more attached to their original opinion.

DECEMBER 1, 1799.

### *New Medical Publications in July.*

A Deſcriptive Account of a new method of treating old ulcers of the legs: By THOMAS BAYNTON, Surgeon of Briſtol. 8vo. ſecond edition. *Hurſt.*

A Caſe of Diabetes, with an Hiſtorical Sketch of that Diſeaſe: By THOMAS GIRDLESTONE, M. D. 8vo. Three Shillings. *Robinſons.*

Practical Obſervations on the Cure of Wounds and Ulcers on the Legs, *without reſt:* By THOMAS WHATELY, Surgeon. 8vo. Seven Shillings. *Cadell* and *Davies.*

Remarks on ſome of the Opinions of the late Mr. John Hunter, reſpecting the Venereal Diſeaſe: By HENRY CLUTTERBUCK, Surgeon. 8vo. One Shilling and Sixpence. *Booſey.*

An Eſſay on Medical Electricity, demonſtrating its effects, particularly in Female Complaints; with Obſervations on the inefficacy of *Metallic Tractors:* to which are added Outlines of Natural Philoſophy: By C. H. WILKINSON. 8vo. Three Shillings and Sixpence. *Allen.*

### NEW MEDICAL PUBLICATIONS IN GERMANY.

*Annalen der Arzneymittellehre.*—Annals of the Materia Medica. By J. J. ROEMER, M. D. Vol. 2d. *Leipzig* Schaefer.

*Verſuch einer Zeichenlehre,* &c.—An Attempt to eſtabliſh a Syſtematic Diagnoſis in the Obſtetric Art: By C. F. ELIAS, M. D. 8vo. 152 p. p. *Marburg.* New Academical Library.

*Mediciniſche Fragmente,* &c.—Medical Fragments derived from experience: By J. G. F. HENNING, M. D. 8vo. 400 pp. *Zerbſt.* Fuchſel.

*Beytræge zur gerichtlichen Arzneykunde,* &c.—Contributions to Medical Juriſprudence: By T. G. A. ROOSE, Prof. 8vo. In Numbers. *Braunſchweig.* Acad. Library.

*Chemiſche Receptirkunſt,* &c. The Art of writing chemico-medical preſcriptions; or a Pocket-book for medical practitioners, who, in preſcribing medicines, wiſh to avoid the errors ariſing from improper chemical combinations in pharmacy: By J. B. TROMMSDORFF, Prof. of Chemiſtry, and Apothecary at Erfurt. Second edition, improved and enlarged. 8vo. 350 pp. *Erfurt.* Bayer and Maring.

### TO CORRESPONDENTS.

We have received a letter dated July 6th, and ſigned W. R. Medicus; which contains ſuch *extraordinary* information on the ſubject of inoculating the *Cow-pox*, as cannot be publiſhed without the real name of its author. The new method of communicating the variolous matter; the ſudden eruption which took place within 48 or 50 hours from the period of communicating the infection; and the very unprecedented mortality among the firſt patients; are circumſtances ſo novel and ſtriking that we cannot venture to lay them before our readers, without more ſubſtantial authority.

The paper with the motto, "*Scire tuum nihil eſt ſine anatomiâ,*" we ſhall have no objection to inſert in our next Number, if the induſtrious author will abridge it, or at leaſt allow us to omit the "Curſory Obſervations;" as his caſes are ſufficiently intereſting, without introductory matter.

The communication on the ſubject of Quackery, ſigned S. M. July 12th. has been neceſſarily delayed, on account of its ſuppoſed libellous tendency, but will be given in our next.

We alſo acknowledge the letter we have received from a Correſpondent, at Birmingham, who ſigns W. B. July 15th.—As it however contains a medical caſe which occurred many years ago, we ſhall conſider, whether we can with propriety inſert it in a future number.

The remarkable Caſe of *Hydrocephalus internus*, communicated by Mr. I. H. dated July 16th, came too late for the preſent, but ſhall certainly appear in our next Number.

The "Obſervations on the animal nature and properties of vital power," by R.K. dated July 17th. likewiſe arrived too late:—we have not had leiſure to conſider the merits of that eſſay, as it is on a ſubject which, though abſtruſe, is intereſting for the plurality of medical readers.

The valuable Caſes of Inoculation for the Cow-pox, tranſmitted to us by our Correſpondent at Mancheſter, will appear in the next Number, for want of room in the preſent.

We are likewiſe obliged to a Correſpondent at Stockport, who has favoured us with a letter reſpecting the uſe of muſk and ſalt of hartſhorn, in gangrenes and mortifications; a medicine originally propoſed by Dr. Darbey—the particulars of which the reader will find in Mr. Simmons's Letter, in this Number.

